# Identification of indocyanine green as a STT3B inhibitor against mushroom α-amanitin cytotoxicity

**DOI:** 10.1038/s41467-023-37714-3

**Published:** 2023-05-16

**Authors:** Bei Wang, Arabella H. Wan, Yu Xu, Ruo-Xin Zhang, Ben-Chi Zhao, Xin-Yuan Zhao, Yan-Chuan Shi, Xiaolei Zhang, Yongbo Xue, Yong Luo, Yinyue Deng, G. Gregory Neely, Guohui Wan, Qiao-Ping Wang

**Affiliations:** 1 Laboratory of Metabolism and Aging, School of Pharmaceutical Sciences (Shenzhen), Shenzhen Campus of Sun Yat-sen University, Shenzhen, PR China; 2 Department of Pathology, The First Affiliated Hospital, Sun Yat-sen University, Guangzhou, PR China; 3 Obesity and Metabolic Disease Research Group, Diabetes and Metabolism Division, Garvan Institute of Medical Research, Darlinghurst, Sydney Australia; 4 School of Pharmaceutical Sciences, Sun Yat-sen University, Guangzhou, PR China; 5 School of Pharmaceutical Sciences (Shenzhen), Shenzhen Campus of Sun Yat-sen University, Shenzhen, PR China; 6 Dr. John and Anne Chong Laboratory for Functional Genomics, Charles Perkins Centre and School of Life & Environmental Sciences, The University of Sydney, Sydney, NSW Australia

**Keywords:** Toxicology, Virtual screening, Drug development

## Abstract

The “death cap”, *Amanita phalloides*, is the world’s most poisonous mushroom, responsible for 90% of mushroom-related fatalities. The most fatal component of the death cap is α-amanitin. Despite its lethal effect, the exact mechanisms of how α-amanitin poisons humans remain unclear, leading to no specific antidote available for treatment. Here we show that STT3B is required for α-amanitin toxicity and its inhibitor, indocyanine green (ICG), can be used as a specific antidote. By combining a genome-wide CRISPR screen with an in silico drug screening and in vivo functional validation, we discover that N-glycan biosynthesis pathway and its key component, STT3B, play a crucial role in α-amanitin toxicity and that ICG is a STT3B inhibitor. Furthermore, we demonstrate that ICG is effective in blocking the toxic effect of α-amanitin in cells, liver organoids, and male mice, resulting in an overall increase in animal survival. Together, by combining a genome-wide CRISPR screen for α-amanitin toxicity with an in silico drug screen and functional validation in vivo, our study highlights ICG as a STT3B inhibitor against the mushroom toxin.

## Abstract (web summary)

There is currently no specific antidote for death cap mushroom poisoning treatment. Here, the authors identify STT3B as a druggable target and show that indocyanine green is a STT3B inhibitor that can block α-amanitin toxicity in cell lines, liver organoids and mice.

## Introduction

Mushroom poisoning is the main cause of mortality in food poisoning incidents worldwide^1,2^. A total of 10,036 exposure events, resulting in 38,676 illnesses and 788 deaths, were reported between 2010 and 2020 in China^3^. Among all poisonous mushrooms, death caps (*Amanita phalloides*) are responsible for more than 90% of death^4^. Amatoxin poisoning is commonly associated with poor outcomes, mainly owing to the irreparable acute failure of the liver or kidney^5,6^.

α-Amanitin (AMA) is one of the most toxic amatoxins. The toxic effects of AMA on humans are considered to be associated with the inhibition of RNA polymerase II (RNAP II)^7^, leading to the production of tumor necrosis factor-α (TNFα)^8^, oxidative stress^9^, and apoptosis^10^. Traditional therapies are often limited to the non-specific decontamination of toxins along with symptomatic and supportive care^5^. During the past decades, several clinical drugs including silybin and penicillin have shown potent therapeutic efficacy on human amatoxin poisoning^11^, although the exact mechanisms of action remain unclear^12^. Moreover, polymyxin B, identified as a potential RNAP II inhibitor in a virtual docking, has been shown to block AMA toxicity in mice^5^. However, specific antidotes targeting specific proteins that play critical roles in AMA toxicity are unavailable since a complete molecular understanding of AMA cytotoxicity is lacking.

Recently, pooled clustered regularly interspaced short palindromic repeats (CRISPR) screens have accelerated our molecular understanding of molecular mechanisms controlling cell death^13,14^. These high-throughput CRISPR screens have been widely utilized to identify genes or pathways involved in drug resistance^15–17^, mechanisms of bacteria toxin^18–20^, or viral infection^21–24^. Furthermore, we have used this approach to dissect the molecular mechanisms of deadly jellyfish venom leading to a potent antidote for jellyfish toxicity^25^.

In this study, we want to establish a systematic framework to develop antidotes by combining the identification of novel drug targets using a genome-wide CRISPR screen and a virtual screen of FDA-approved drugs. We first performed a genome-wide CRISPR loss-of-function screen to identify genes and pathways involved in AMA cytotoxicity. We found that several novel pathways including N-Glycan biosynthesis and cholesterol metabolism were involved in AMA-induced cell death. We further showed that N-Glycan biosynthesis and its catalytic enzyme STT3B were required for AMA toxicity. By combining these data with in silico screening of FDA-approved drugs and follow-up functional validation, we successfully identified ICG as a potential STT3B inhibitor. We further demonstrated that ICG could block AMA-induced cell death in vivo and in vitro, suggesting ICG may have utility in treating AMA/death cap poisoning.

## Results

### A high-throughput CRISPR screen to identify genes and pathways required for AMA-induced cell death

**Fig. 1:**
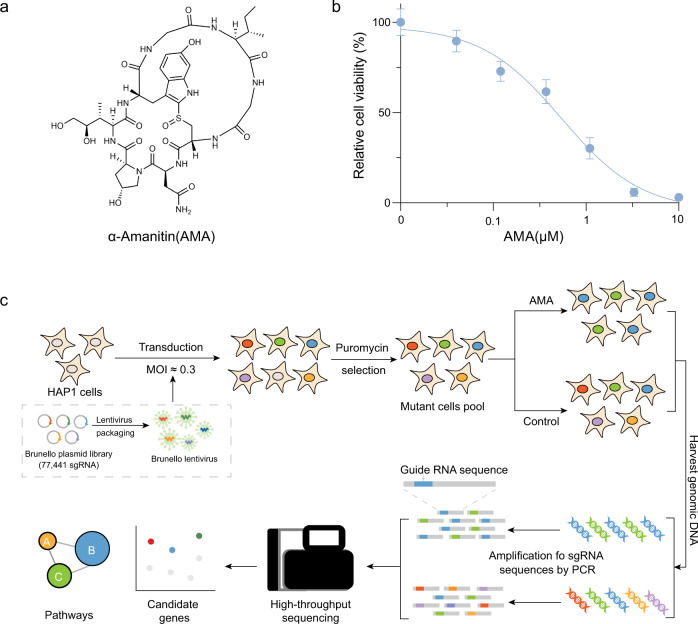
A workflow of a genome-wide CRISPR-Cas9 knockout screen for AMA toxicity. **a** Chemical structure of AMA. **b** HAP1 cells were treated with vehicle or different concentrations of AMA for 72 h, and cell viability was determined by CCK8 assays (*n* = 3 biological replicates). Data are presented as mean ± SD and are representative of three independent experiments. **c** The workflow of genome-wide CRISPR loss-of-function screening. Source data are provided as a Source Data file.

*Amanita phalloides* is a common cause of death from food poisoning, primarily due to its production of AMA^26^ (Fig. 1a). To identify the key genes and pathways required for AMA-induced cell death, we carried out a genome-wide loss-of-function screen that targets a total of 19,114 genes using the human CRISPR knockout pooled library (Brunello)^27^. We performed our screening using haploid cell line HAP1, which has been extensively used to investigate the mechanisms of drug resistance^15^, toxicology^25,28^, synthetic lethality^29^, and virus infection^30^. Before the screening, we first determined the 50% inhibitory concentration (IC_50_) of AMA for HAP1 cells (Fig. 1b). We then transduced HAP1 cells with Brunello library at a low multiplicity of infection (MOI ≈ 0.3) to make sure that most HAP1 cells receive only one genetic perturbation (Fig. 1c). Meanwhile, the coverage of >500 cells expressing each 77,441 sgRNA was ensured. Transfected cells were then selected with 1 μg/mL puromycin for 7 days. Mutant pools of cells were then challenged with a dose of 1.5 μM AMA for 7 days, and the genomic DNA was extracted from the surviving cells for deep sequencing.

**Fig. 2:**
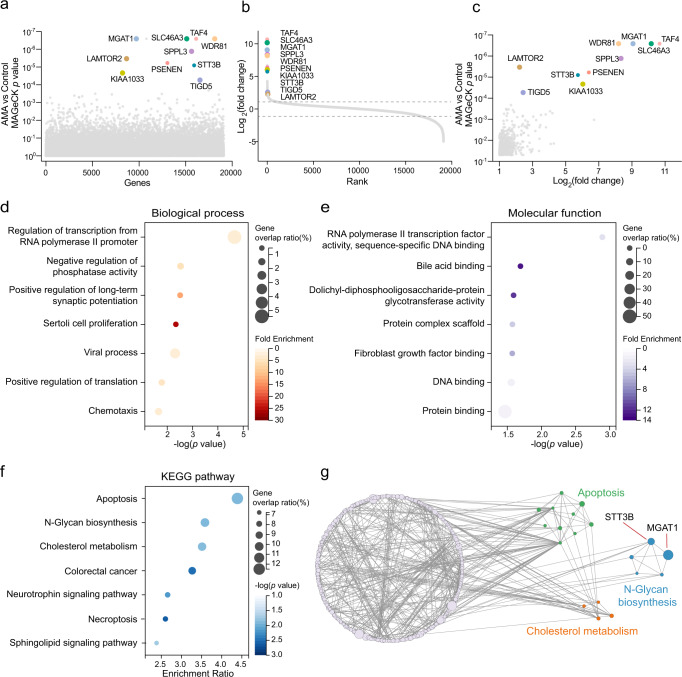
The CRISPR screen identifies genes and pathways required for AMA toxicity. **a** Bubble plot of *p* value from AMA screen. **b** Distribution of Log_2_ fold change (LFC, AMA treatment versus control) for each gene. **c** The *p* value and LFC of significant 559 genes. The top 10 genes were highlighted and marked. **d**, **e** GO terms enrichment analysis of these significant hits for biological process **d** and molecular function **e**. **f** KEGG pathway analysis of these significant hits. **g** Genetic interaction network of the significant 559 genes. The node size represents the -Lg (RRA score). Source data are provided as a Source Data file.

After model-based analysis of genome-wide CRISPR/Cas9 knockout (MAGeCK) analysis, we have identified hundreds of genes that were associated with AMA-induced cell death (Fig. 2a, b, Supplementary Data 1). A subset of sgRNAs targeting 559 genes was significantly changed (*p* < 0.05 and |LFC | > 1) in the AMA-treated cells when compared to the untreated controls, indicating that these genes were involved in AMA toxicity (Fig. 2c). We first performed the bioinformatic analyses of gene ontology (GO) and KEGG on these altered genes. As expected, “Regulation of transcription from RNA polymerase II promoter” was the top enriched in the biological process (Fig. 2d), and “RNA polymerase II transcription factor activity sequence-specific DNA binding” also was the top enriched in the molecular function classifier (Fig. 2e). This is consistent with our previous understanding that AMA acts by inhibiting RNA polymerase II^31^. Several KEGG pathways including apoptosis, N-Glycan biosynthesis and cholesterol metabolism were also enriched in AMA-induced cell death (Fig. 2f). We further employed a network propagation approach to better understand the gene network between these enriched KEGG pathways (Fig. 2g). Among these enriched processes or pathways, RNA polymerase II transcription and apoptosis^10,32^ have been linked to AMA toxicity, but N-glycan biosynthesis and cholesterol metabolism have not yet been reported, suggesting these two pathways may play a vital role in AMA toxicity. Together, our screen has identified novel pathways required for AMA-induced cell death.

### N-Glycan biosynthesis is required for AMA-induced cell death

**Fig. 3:**
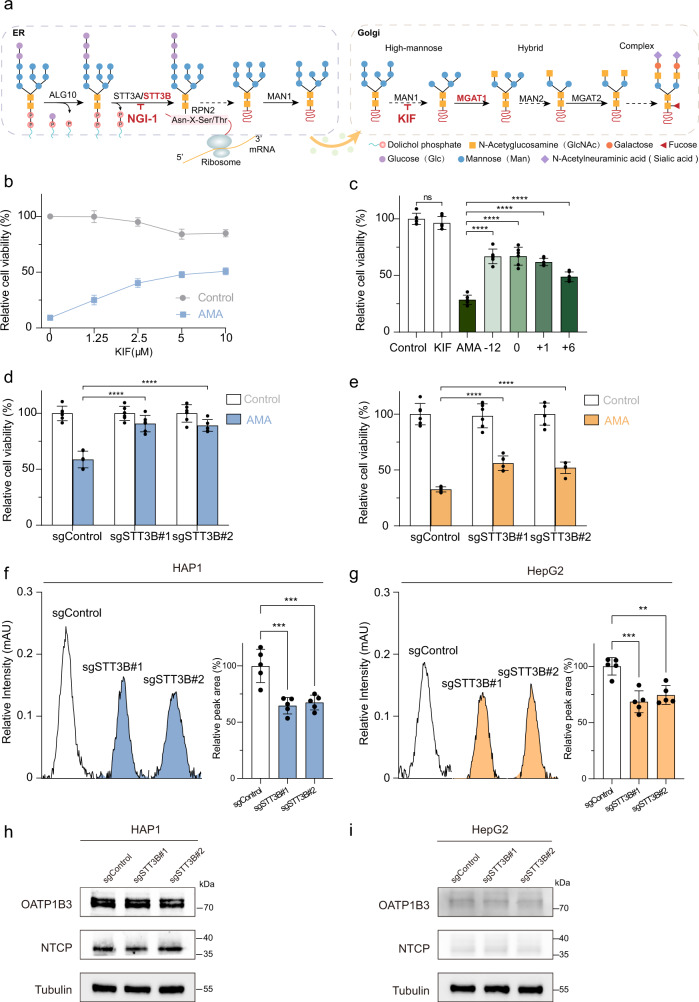
N-Glycan biosynthesis is essential for AMA-induced cell death. **a** A simplified overview of N-glycan biosynthesis. **b** KIF blocked AMA toxicity. HAP1 cells were pre-treated with KIF for 12 h and then treated with AMA (3 μM) for 48 h (*n* = 3 biological replicates). **c** Both pre-treatment and post-treatment with KIF protect HAP1 cells against AMA (3 μM) (*n* = 6 biological replicates). ^ns^*p* = 0.9117, ****p < 0.0001. The statistics were assessed using one-way ANOVA followed by Tukey’s multiple comparisons test. **d**, **e** Depletion of *STT3B* conferring resistance to AMA in HAP1 **d** and HepG2 **e** cells (*n* = 6 biological replicates). ****p < 0.0001. The statistics were assessed using one-way ANOVA followed by Dunnett’s multiple comparisons test. **f**, **g** The detection of AMA entrance into cells. The representative peak and relative peak area (*n* = 5 biological replicates) of AMA in different sgRNA *STT3B* knockout HAP1 **f**, and HepG2 **g** cells. ***p = 0.0003, ***p = 0.0006; ***p = 0.0002, **p = 0.0010. The statistics were assessed using one-way ANOVA followed by Dunnett’s multiple comparisons test. **h**, **i** The expression of OATP1B3 and NTCP in different sgRNA *STT3B* knockout HAP1 **h** and HepG2 **i** cells. Data are presented as mean ± S.D.  and are representative of three independent experiments. Source data are provided as a Source Data file.

The N-glycan biosynthesis pathway is of particular interest since its key components *STT3B* and *MGAT1* were enriched in the top 10 ranking genes. N-glycans on glycoproteins serve as one of the most important co- and post-translational protein modifications in eukaryotic cells^33^ and have multiple biological functions, such as cell adhesion, intracellular signaling, homeostasis, and inflammation^34,35^. The N-glycan biosynthesis occurs in the endoplasmic reticulum (ER) and Golgi apparatus and requires a series of processes catalyzed by multiple enzymes including ALG10, STT3A/3B, MAN1, MGAT1, MAN2, and MGAT2 (Fig. 3a)^36^. We found that the counts for sgRNA targeting *ALG10, STT3A/3B, RPN2*, and *MGAT1* were enriched in AMA-treated cells (Supplementary Fig. 1a–e) and *STT3B* and *MGAT1* were enriched in the top 10 hits. To validate the role of N-glycan biosynthesis in AMA-induced cell death, we first utilized kifunensine (KIF), a potent small alkaloid inhibitor, to pharmacologically inhibit MAN1 activity to block the synthesis of N-glycans^37,38^. As anticipated, KIF-treated cells exhibited resistance to AMA-induced death in HAP1 cells (Fig. 3b). Importantly, KIF also inhibited cell death in HAP1 cells pretreated with AMA for 6 hours (Fig. 3c).

STT3B is an upstream component of the N-glycan biosynthesis pathway^39^. STT3B and STT3A form the oligosaccharyltransferase (OST) complex that catalyzes posttranslational glycosylation in ER^40,41^. To further confirm the role of *STT3B* in AMA toxicity, we generated *STT3B* knockout HAP1 and HepG2 cell lines using CRISPR-Cas9 technology. Accordingly, STT3B mRNA expression was severely reduced in both cells (Supplementary Fig. 2a, b). The depletion of *STT3B* resulted in elevated resistance to AMA-induced cell death in HAP1 cells (Fig. 3d) and HepG2 cells (Fig. 3e). We further confirmed these results in HepG2 cells with STT3B knockdown using short hairpin RNA (shRNA) (Supplementary Fig. 2c). Since STT3B works together with STT3A in protein glycosylation, we asked whether the depletion of STT3A in *STT3B* knockout cells could cause more resistance to AMA cytotoxicity. When STT3A was knocked down in *STT3B* knockout HepG2 cells, these cells gained almost complete resistance to AMA-induced cell death (Supplementary Fig. 2d-e), suggesting that the partial resistance of *STT3B* knockout HepG2 to AMA was due to the expression of STT3A OST complex, which is functionally redundant to the STT3B OST complex^40^. Moreover, we used a small molecule N-linked glycosylation inhibitor 1 (NGI-1) to block the activity of OST^42,43^. NGI-1 can inhibit AMA-induced cell death at 2.5 μM, however, NGI-1 itself was toxic to cells at 5-20 μM (Supplementary Fig. 2f), suggesting NGI-1 could not be used as an antidote to AMA toxicity.

We next asked whether glycan biosynthesis affects the entrance of AMA into cells. The intracellular AMA content was quantified with an established high-performance liquid chromatographic (HPLC) assay (Supplementary Fig. 3). The depletion of STT3B significantly decreased the entrance of AMA into HAP1 cells (Fig. 3f) and HepG2 cells (Fig. 3g). Meanwhile, *STT3B* knockout does not affect the expression of OATP1B3 and NTCP, which are AMA transporters in those cells (Fig. 3h, i). The inhibition of glycosylation may trigger stress responses in the ER and Golgi^44–46^, however, *STT3B* knockout did not cause stress responses in both ER and Golgi (Supplementary Fig. 4). Together, all data suggests that *STT3B* is required for AMA-induced cell death and affects the entrance of AMA into cells.

### In silico screen of FDA-approved molecules for STT3B inhibitor

**Fig. 4:**
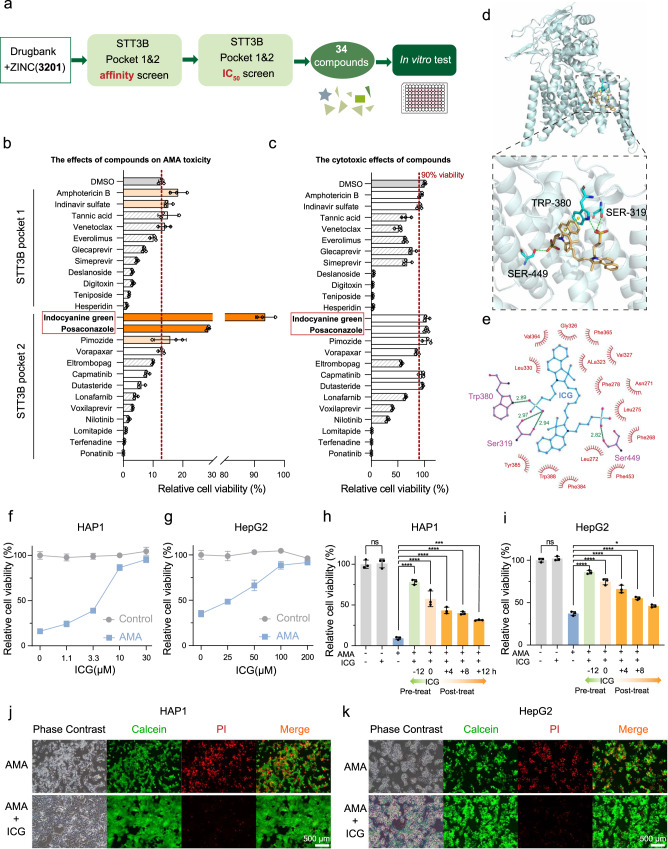
In silico screen of FDA-approved molecules for STT3B inhibitor. **a** Schematic design of in silico screen to identify STT3B inhibitors. Minimized affinity screen and NNScore2 screen were subsequently performed. **b** Relative cell viability of HAP1 cells pretreated for 12 h with the indicated small molecule inhibitors at 10 μM and then treated with 3 μM AMA (*n* = 3 biological replicates). **c** Relative cell viability of HAP1 cells treated with the indicated small molecule inhibitors at 10 μM for 60 h (*n* = 3 biological replicates). **d** 3D Overview and close-up views of binding sites of STT3B and ICG (generated by PyMOL). ICG is shown in light orange. STT3B residues interacting with ICG are shown in cyan. Hydrogen bonds are shown in dashed green lines, and pi-pi stacking is shown in dashed yellow lines. **e** 2D STT3B-ICG interaction diagrams (generated by LigPlot + ). ICG is shown in blue, hydrogen-bonding residues are shown in purple, and hydrogen bonds are shown in green dotted lines, the spoked arcs represent residues making nonbonded contacts with ICG. **f**, **g** The pretreatment of ICG reduces AMA-induced cell death. Cells were pre-treated with ICG for 12 h and then treated with AMA (3 μM for HAP1 and 5 μM for HepG2) for 48 h (*n* = 3 biological replicates). **h**, **i** The pre-treatment and post-treatment of ICG (10 μM for HAP1 and 100 μM for HepG2) protected HAP1 (3 μM) and HepG2 (5 μM) cells from AMA-induced cell death (*n* = 3 biological replicates). ^ns^*p* > 0.9999, ****p < 0.0001, ***p = 0.0007; ^ns^*p* = 0.9840, ****p < 0.0001, *p = 0.0181. The statistics were assessed using one-way ANOVA followed by Tukey’s multiple comparisons test. **j**, **k** Calcein/PI viability assay of HAP1 cells and HepG2 cells pretreated with ICG for 12 h and then treated with AMA at 3 μM for HAP1 cells **j** and at 5 μM for HepG2 cells **k**. Scale bars are 500 μm. Data are presented as mean ± SD.  and are representative of three independent experiments. Source data are provided as a Source Data file.

Since the blockage of N-glycan biosynthesis can prevent AMA-induced cell death, the STT3B inhibitors would be potential antidotes for treating AMA toxicity. So far, no FDA-approved molecule has been reported to specifically inhibit STT3B. Therefore, we performed an in silico screen of FDA-approved molecules to look for potential STT3B inhibitors. The FDA molecules libraries (ZINC and Drugbank) including a total of 3201 compounds were used for virtual screening for STT3B inhibitors. There were two putative binding pockets in STT3B. After in silico screen, a total of the top 34 compounds were selected for in vitro cellular validation (Fig. 4a, Supplementary Data 2). Due to the unavailability of some compounds, only 24 compounds were tested for cellular protection against AMA toxicity. Of all tested drugs, ICG and posaconazole could significantly prevent cell death in HAP1 cells (Fig. 4b) without additional cytotoxicity to cells (Fig. 4c). ICG provided almost full protection against AMA cytotoxicity. In molecular docking, ICG binds to STT3B potentially blocking the catalytic activity. ICG has several contacts with STT3B **(**Fig. 4d, e) including the three oxygen of two sulfobutyl moieties formed three hydrogen bonds with side chains of Ser319, Trp380 and Ser449. In addition, the benzene ring of benzoindolyl moiety in ICG participated in an offset face-to-face pi-stack with the side chain of Trp380 in STT3B. The inhibitory effect of ICG is mediated by the occupation of the entrance of the STT3B substrate binding pocket.

### ICG alleviates AMA toxicity in cells and liver organoids

ICG, a fluorescent iodide dye, has been approved by FDA as a diagnostic reagent in humans since 1956 and is now widely used in ocular angiography and hepatic function assessment^47^. ICG can be rapidly cleared by hepatocytes^47^ and ICG has no obvious side effects at a standard single dose of 0.5 mg/kg (50% lethal dose is 60-80 mg/kg for mice)^48^. Therefore, we further assessed the therapeutic effect of ICG on AMA toxicity.

ICG pretreatment provided a dose-dependent reduction in cell death in both HAP1 cells (Fig. 4f) and HepG2 cells (Fig. 4g). Furthermore, ICG treatments also significantly inhibited cell death in AMA-pretreated HAP1 cells (Fig. 4h) and HepG2 cells (Fig. 4i). To further confirm that ICG can block AMA toxicity, we monitor cell death using Calcein/propidium iodide (PI) staining and again found cells pre-treated with ICG were much more resistant to AMA-induced cell death (Fig. 4j, k).

**Fig. 5:**
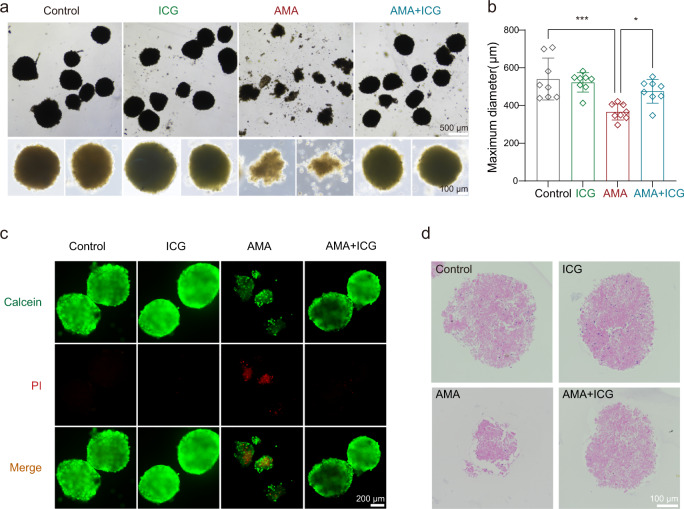
ICG alleviates AMA toxicity in mice liver organoids. The Organoids were treated with AMA (3 μM) and/or ICG (10 μM) for 3 days. **a** Representative images of mouse liver organoids. **b** Maximum diameter of organoids (*n* = 8 randomly selected organoids in each figure). ***p = 0.0002, *p = 0.0248. The statistics were assessed using one-way ANOVA followed by Tukey’s multiple comparisons test. **c** Calcein/PI viability assay of organoids. **d** Representative H&E images of organoids. Data are presented as mean ± SD.  and are representative of three independent experiments. Source data are provided as a Source Data file.

Moreover, we established a mouse liver organoid model for further evaluation of the ICG therapeutic effect. Consistent with our observations in HAP1 and HepG2 cells, ICG could effectively block AMA’s cytotoxic effect on these liver organoids. ICG-treated organoids were more tightly connected and larger than the vehicle-treated organoids (Fig. 5a, b). We also observed that ICG treatment significantly prevented AMA-induced cell death by Calcein/PI staining assay (Fig. 5c). The ICG treatment effect was also observed by hematoxylin and eosin (H&E) staining and ICG blocked the AMA toxicity on organoids (Fig. 5d). Together, combining functional CRISPR screening with in silico drug prediction is a viable pipeline to quickly identify novel toxin antidotes, and here we show ICG is a potential STT3B inhibitor that can prevent AMA-induced cell death.

### ICG prevents AMA-induced cell death by inhibiting STT3B activity

**Fig. 6:**
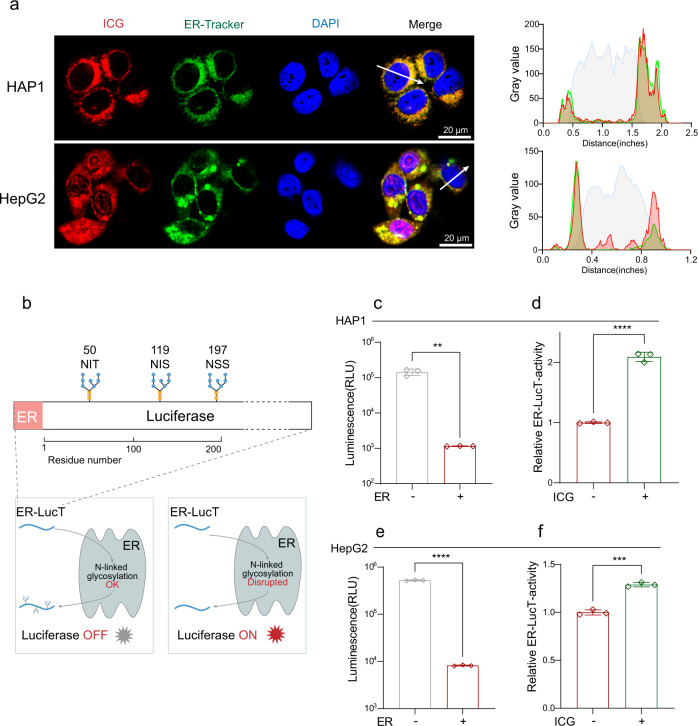
ICG prevents cells from AMA-induced cell death by inhibiting STT3B activity. **a** Intracellular co-localization of ICG and ER in HAP1 and HepG2 cells and the corresponding fluorescence intensity profiles across the cell along the direction of arrow. **b** A scheme of ER-LucT reporter system, the disruption of luciferase glycosylation turn on luminescence. Luc, luciferase. **c** N-glycosylation of Luc reduced the Luc activity in HAP1 cells (*n* = 3 biological replicates). **p = 0.0012 **d** The blockage of N-glycosylation of Luc increased the Luc activity by treatment with 10 μM ICG. The ER-LucT-activity was normalized to the vehicle control (*n* = 3 biological replicates). ****p < 0.0001. **e** N-glycosylation of Luc reduced the Luc activity in HAP1 cells (*n* = 3 biological replicates). ****p < 0.0001. **f** The blockage of N-glycosylation of Luc increased the Luc activity by treatment with 100 μM ICG HepG2. The ER-LucT-activity was normalized to the vehicle control (*n* = 3 biological replicates). ***p = 0.0001. Data are *p*resented as mean ± SD and are representative of three independent experiments. The statistics were all assessed using two-tailed unpaired *t* test. Source data are provided as a Source Data file.

To test whether ICG can interact with STT3B in cells, we detected the intracellular localization of ICG. ICG can produce green fluorescence. We found that ICG was co-localized with ER in HAP1 cells and this colocalization was confirmed in HepG2 cells (Fig. 6a), suggesting ICG acts in the ER, where STT3B is also localized. To further test whether ICG can inhibit STT3B activity, we used a bioluminescent reporter namely ER-LucT to assess STT3B activity by an ER-LucT reporter system^42,43^. This system consists of a modified luciferase (Luc) with an ER translation sequence and three (T) potential glycosylation sites^49^. The N-glycosylation of the modified luciferase inhibits its activity and reduces bioluminescence (Fig. 6b). We first tested the ER-LucT reporter system in HAP1 cells. The luminescence was significantly decreased in cells with the ER-LucT, indicating luciferase activity was inhibited (Fig. 6c). As expected, consistent with NGI-1 (Supplementary Fig. 5a), ICG increased the activity of ER-LucT by decreasing the N-glycosylation mediated by STT3B (Fig. 6d). Similar results were also observed in HepG2 cells (Figs. 6e, f, Supplementary Fig. 5b). Together, ICG prevents cells from AMA-induced cell death by inhibiting STT3B activity.

### ICG is an effective antidote for treating AMA toxicity in mice

**Fig. 7:**
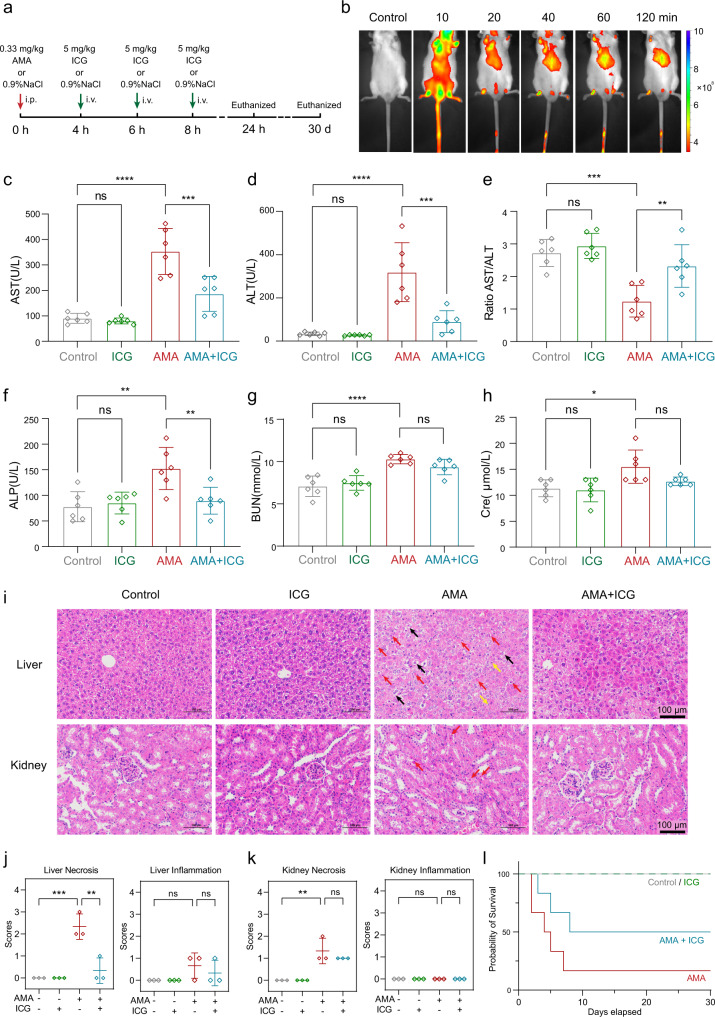
ICG is an effective antidote for AMA toxicity in mice. **a** The scheme of the mouse study. AMA was i.p. injection at 0.33 mg/kg and ICG intravenously injected at 4 h, 6 h and 8 h with a dose of 5 mg/kg. The mice were euthanized at 24 h and 30th day. **b** NIR fluorescence images of mice at different time points after intravenous injection of ICG. **c**–**h** Plasma levels of AST, ALT, the ratio of AST/ALT, ALP, BUN, Cre in different groups (*n*  =  6 biological replicates). **c** ^ns^*p* = 0.9889, ****p < 0.0001, ***p = 0.0004; **d** ^ns^*p* = 0.9986, ****p < 0.0001, ***p = 0.0001; **e** ^ns^*p* = 0.8764, ***p = 0.0002, **p = 0.0060; **f** ^ns^*p* = 0.9748, ***p = 0.0020, **p = 0.0091; **g** ^ns^*p* = 0.8776, ****p < 0.0001, ^ns^*p* = 0.3169; **h** ^ns^*p* = 0.8776, *p = 0.0163, ^ns^*p* = 0.3169. **i** H&E staining of liver and kidney of mice in different treatments. Cellular edema (black arrow), inflammatory cells (yellow arrow), and necrosis (red arrow) were shown. Scale bars are 100 μm. **j**, **k** Pathological score of liver and kidney in different treatments (*n* = 3 biological replicates). **j** ***p = 0.0005, **p = 0.0015; ^ns^*p* = 0.2641, ^ns^*p* = 0.7538; **k** **p = 0.0021, ^ns^*p* = 0.5252; the sam*p*les all have a standard error of zero. **l** Survival curves of mice with different treatments (*n* = 6 biological replicates). Data are presented as mean ± SD. The statistics were assessed using one-way ANOVA followed by Tukey’s multiple comparisons test. Source data are provided as a Source Data file.

We next tested the efficacy of ICG as an AMA antidote in animals. To mimic actual human AMA toxicity^50,51^, ICG was given to mice that were pre-treated with an intraperitoneal (i.p.) AMA for 4 hours at 0.33 mg/kg as previously reported^5,52^. Three consecutive administrations of ICG at 5 mg/kg, equal to approximately 0.5 mg/kg in humans^53^, were intravenously(i.v.) injected into mice at 4 h intervals (Fig. 7a). ICG distribution was monitored by near-infrared (NIR)^54^. After injection, ICG was rapidly distributed all over the body and mainly concentrated in the liver after 2 h (Fig. 7b**)**, which is consistent with previous observations that ICG is rapidly cleared from the plasma and was selectively taken by liver^55^.

As the liver and kidney are the major AMA-targeting organs, we assessed the protective effects of ICG on the AMA-exposed liver and kidney. The damages in the liver and kidney were evaluated by measuring plasma biomarkers and histopathological analyses. After AMA treatment, liver biomarkers aspartate aminotransferase (AST), alanine aminotransferase (ALT), and alkaline phosphatase (ALP) were significantly increased (Fig. 7c, d, f) and the AST/ALT ratio was markedly decreased (Fig. 7e), indicating that livers were severely injured by AMA. Importantly, ICG treatment significantly reduced the levels of AST, ALT, and ALP, indicating that ICG can block the liver damage caused by AMA (Fig. 7c–f). Similar results were observed for kidneys, with ICG treatment significantly reducing renal biomarker blood urea nitrogen (BUN) and creatinine (Cre) in the AMA-treated mice (Fig. 7g, h).

ICG treatment also significantly reduced inflammatory cell infiltration and necrosis in the liver of AMA-treated mice (Fig. 7i). These data were quantified, and histological scores confirmed that ICG can suppress AMA-induced liver necrosis (Fig. 7j) and similar results were observed for AMA and ICG-treated kidneys (Fig. 7i, k). Moreover, we performed a long-term (30 days) survival assay to evaluate if ICG could also protect from AMA-induced death, finding that ICG treatment significantly improved the survival of AMA-treated mice (Fig. 7l). Moreover, we did not observe obvious side effects in the ICG-only mice in both short- and long-term studies, demonstrating that this dose of ICG is safe for treating AMA poisoning in mice.

We also assessed longer time intervals (up to 12 h) between AMA injection and ICG treatment in a mouse model. Our results showed that the time intervals between AMA injection and ICG delivery were important for the ICG treatment effect. The intervals of 8 h and 12 h limited the ICG treatment effect, and intervals of 1 h and 4 h had better therapeutic efficacies (Supplementary Fig. 6). This indicates that ICG is needed to be given as early as possible when AMA poisoning occurs.

**Fig. 8:**
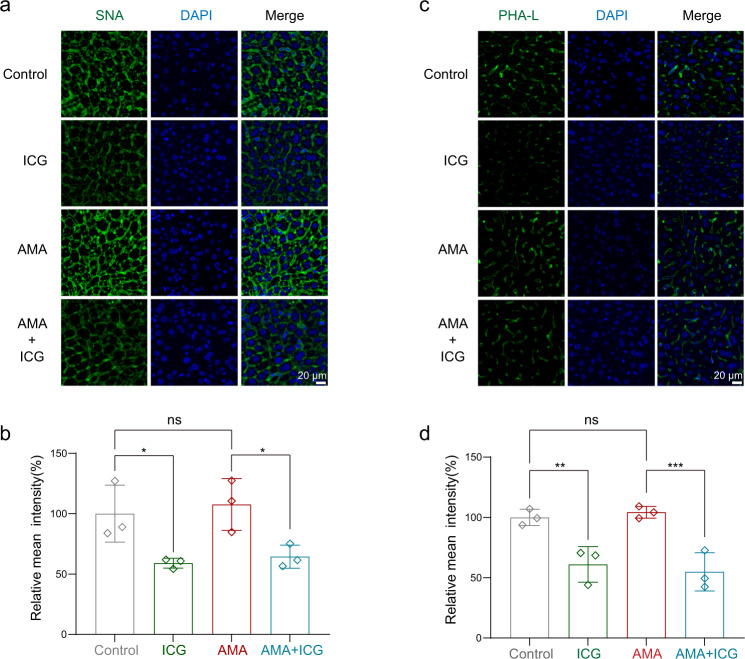
ICG disrupts glycation in livers in vivo. The lectin staining assay was used to analyze the glycation of liver sections. The mice were administrated with AMA at 0 h and three consecutive administrations of ICG were injected at 4 h, 6 h, 8 h, and all mice were euthanized at 24 h. The liver sections were stained with SNA for sialylated glycans and PHA-L for complex glycans. **a**, **b** Representative images of SNA binding to complex glycans **a** and semiquantitative evaluation for SNA **b** by Image J (*n* = 3 biological replicates). *p = 0.0174, ^ns^*p* = 0.5946, *p = 0.0136. **c**, **d** Representative images of sialylated glycans stained by PHA-L **c** and semiquantitative evaluation **d** by Image J (*n* = 3 biological replicates). **p = 0.0033, ^ns^*p* = 0.6628, ***p = 0.0008. Data are presented as mean ± SD. The statistics were assessed using a one-way ANOVA test. Source data are provided as a Source Data file.

To test the inhibitory effect of ICG on protein glycan synthesis in animals, we performed lectin staining to assess in vivo glycation. We applied fluorescein-sambucus nigra agglutinin (SNA) and fluorescein-phaseolus vulgaris leucoagglutinin (PHA-L) to stain sialylated glycans and complex glycans, respectively, as previously reported^56^. As expected, SNA and PHA-L can label the glycated proteins localizing on the plasma membrane of liver cells. ICG could significantly reduce the fluoresces of SNA (Fig. 8a, b) and PHA-L (Fig. 8c, d) staining when compared to the vehicle, suggesting ICG could effectively disrupt the glycation in vivo. Together, these data show that ICG is an effective antidote for treating AMA toxicity in mice.

## Discussion

**Fig. 9:**
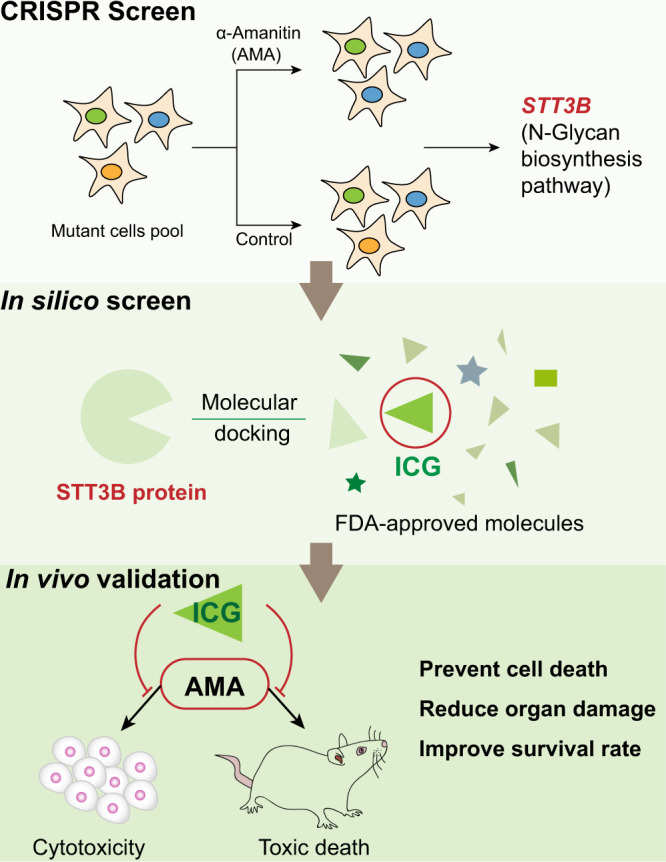
Identification of ICG as a STT3B inhibitor against mushroom AMA cytotoxicity. A genome-wide CRISPR screen against mushroom AMA cytotoxicity has identified STT3B, a key enzyme in the N-glycan biosynthesis pathway, as a druggable target for preventing AMA-induced cell death. After an in silico drug screen with an FDA-approved library, ICG was identified as a specific inhibitor for STT3B. Eventually, ICG could effectively block AMA cytotoxicity in vitro and in vivo.

Our unbiased genome-wide CRISPR screen has identified that both STT3B and the N-glycan biosynthesis pathways are required for AMA toxicity, and these data were validated both genetically and pharmacologically. Furthermore, by combining our CRISPR screening with in silico screening of druggable targets, we uncovered an FDA-approved compound ICG that could serve as a novel potential antidote to relieve AMA toxicity both in cells and in animals (Fig. 9). Our findings highlight that a combined approach using genome-wide CRISPR screening coupled with in silico drug prediction can help us quickly identify new antidotes for medically relevant human poisons.

Since consumption of Death Cap (*Amanita phalloides*) leads to a high rate of mortality, there is an urgent need to better understand the molecular toxicology of its major toxic component AMA. This consequently facilitates the discovery of effective antidotes to treat mushroom poisoning. By using a genome-wide CRISPR-Cas9 screen, we have identified several important genes and pathways involved in AMA-induced cell death. To support the ability of the screen to identify key factors, some of the well-known mechanisms involved in AMA toxicity such as apoptosis and RNA polymerase II inhibition were among the pathways and GO terms. One of our top pathways, N-Glycan biosynthesis, was validated both pharmacologically and genetically.

N-linked glycosylation plays a critical role in protein folding and trafficking, which is involved in a large number of biological recognition events^34^. It has been reported that bacterial toxins^37,57^ and viruses^23,58^ bind N-glycans and this interaction facilitates the entry into targeting cells. In this case, we hypothesized that the blockage of N-glycans of AMA transporters may hinder the recognition of AMA, which contributes to the resistance.

STT3B is a key enzyme for N-glycan biosynthesis and it has been considered a therapeutic target for treating cancers^42^. However, no FDA-approved compound has been identified for the inhibition of N-glycan biosynthesis or STT3B. Through in silico screening of FDA-approved molecules, we successfully demonstrated that ICG was a potential STT3B inhibitor for inhibiting N-glycan biosynthesis. ICG is a water-soluble near-infrared fluorescent dye, which has been widely used for decades as a diagnostic agent to measure hepatic functions, cardiac output, and ophthalmic angiography in humans^59,60^. We showed that ICG could effectively prevent AMA-induced cell death and increase resistance to AMA in vitro. The enterohepatic cycle of AMA could significantly influence the clinical course of amatoxin poisoning in humans and animals^61^. Interruption of enterohepatic circulation such as biliary drainage has become an alternative detoxification method^62^. Since ICG can prevent the entrance of AMA, therefore, ICG may block AMA recirculation and prohibit AMA toxicity and this partially explains the ICG’s therapeutic effect.

HepG2 cells have been used for investigating hepatotoxicity^63^ and molecular mechanisms of AMA cytotoxicity^7^. HepG2 cells show a relatively low expression of OATP1B3^64^ and do not express NTCP^65^, while these two proteins are considered as main AMA uptake transporters. Therefore, HepG2 cells present lower sensitivity to AMA cytotoxicity compared to other common laboratory cell lines or primary hepatocytes^66^. Nevertheless, in our study, HepG2 cells still can be killed by AMA and rescued by ICG, suggesting that there are existing unknown transporters responsible for AMA transporting into cells. Interestingly, ICG was also transported into cells by OATP1B3 and NTCP^67^. Our results exclude the possibility that ICG mainly acts by competitive binding with OATP1B3 and NTCP to block AMA’s toxicity. Furthermore, our study showed that ICG has similar protective effects in AMA-treated primary hepatocytes organoids.

In this study, we focused on 4 h interval between AMA injection and ICG treatment since this 4 h interval was the same as that used in previous studies to mimic actual human poisoning and treatment scenarios^5,52,68^. Therefore, this will facilitate us to compare our results with others on how ICG is effective in treating AMA toxicity. ICG has shown great potential for treating AMA poisoning in mice and ICG treatment can significantly attenuate AMA-induced damage in the liver and kidney, the two major AMA-targeting organs, resulting in improved survival. Furthermore, we observed that ICG lose its treatment effect on AMA toxicity when it was delivered 8 and 12 h post AMA injection. This may be because AMA has caused irreversible damage during early hours of cytotoxicity, which is unable to be salvaged by ICG treatment. This suggests that ICG should be given as early as possible during treatment.

Overall, we show that by coupling whole-genome functional genomic characterization with in silico drug prediction, we can rapidly define and then target medically relevant processes.

## Methods

### Ethical statement

All animal experiments were approved by the Institutional Animal Care and Use Committee (IACUS) of Sun Yat-Sen University (Approval number: SYSU-IACUC-2022-000469).

### Cell lines and cell culture

HAP1 cells were obtained from Horizon Discovery. HEK293T and HepG2 cell lines were obtained from the American Type Culture Collection (ATCC). All cell lines were routinely tested with mycoplasma free by Mycoplasma Stain Assay Kit (#C0296, Beyotime) and authenticated by Short Tandem Repeat (STR) profiling. HAP1 cells were cultured in Iscove’s Modified Dulbecco’s Medium (IMDM; Gibco) supplemented with 10% Fetal Bovine Serum (FBS; NEWZERUM) and 1% penicillin-streptomycin (Hyclone). HEK293T and HepG2 cells were cultured in Dulbecco’s modified Eagle’s medium (DMEM, Gibco) supplemented with 10% FBS and 1% penicillin-streptomycin.

### Cell viability assay

Trypsinized cells (1.5 × 10^4^) were seeded in each well of a 96-well plate. After 24 h, various concentrations of compounds were added, and the cells were incubated for an additional 48 or 72 h. KIF(#K919109) and NGI-1(#N873007) were obtained from Macklin. After incubation, the medium was aspirated from each well and 100 μL fresh medium containing a 10% Cell Counting Kit-8 (CCK8, #K1018, APExBIO) was added to the wells and incubated for 2 h at 37 °C. The absorbance was measured at 450 nm using a microplate spectrophotometer (BioTek). The cell survival data of each drug-treated group were normalized to the vehicle group and expressed the survival data as “relative cell viability”. Calcein/PI Cell Viability was assessed according to the protocol of the manufacturer (#C2015M, Beyotime) and was observed under fluorescence microscopy (Nikon).

### Lentivirus production

To generate lentivirus, the Brunello Library (#73179, Addgene) was co-transfected with packaging plasmids pMD2.G(#12259, Addgene) and psPAX2 (#12260, Addgene). Briefly, a T75 flask of 80% confluent HEK293T cells was transfected in Opti-MEM (#31985070, Gibco) using 8.8 μg of the lentiCRISPRv2 plasmid library, 4.4 μg pMD2.G, 6.7 μg psPAX2, and 32 μL of Lipo8000^TM^ (#C0533, Beyotime). Cells were incubated for 8 h and then the media was changed to DMEM with 10% FBS and 1% penicillin-streptomycin. The virus was harvested 48 h post-transfection, the media was replenished, and a second harvest occurred at 72 h post-transfection. Viral supernatants were collected and filtered through a 0.45 μm ultra-low protein binding filter (Merck Millipore). Aliquots were stored at −80 °C.

### Cell transduction using the Brunello library

Infections were performed in a 12-well plate with 2.0 × 10^6^ cells per well. Different volumes of viruses were added to each well. After 24 h, the cells were trypsinized and each well was split into duplicate wells. And one replicate was treated with 1 μg/ml puromycin (#P8230, Solarbio) for 3 days until the no-virus conditions contained no viable cells. Finally, the cell viability was quantified for each condition using a CCK8 assay. The virus volume yielding an MOI of approximately 0.3 was used for next large-scale screening.

### HAP1 cells AMA resistance screen

To ensure the coverage of >500 cells expressing each 77,441 sgRNA at an MOI ≈ 0.3, 1.3 × 10^8^ HAP1 cells were transduced as described above using 12-well plates with 2 × 10^6^ cells per well. Puromycin was added to the cells 24 h post-transduction and maintained for 7 days. Cells were pooled together into larger flasks after 3 days of incubation of puromycin. On day 7, cells were split into treatment conditions in duplicate with a minimum of 4 × 10^7^ cells per replicate. Two replicates were cultured in a complete medium with 1.5 μM AMA (#A4548, APExBIO), and another two replicates were cultured in a regular complete medium. Replicates were either passed, or fresh media was added every 2–3 days. The mutant cell pools were treated by AMA for 7 days and the surviving cells were recovered and harvested for genomic DNA analysis.

### Genomic DNA sequencing

Genomic DNA (gDNA) was isolated using TIANamp Genomic DNA kits (#DP304, TIANGEN) according to the manufacturer’s protocol. The sgRNA sequences were amplified using High-Fidelity 2X PCR Master Mix (#M0541L, NEB). PCR products were gel extracted, quantified, mixed and sequenced using Illumina (PE150) by Novogene Technology (Beijing, China). Enrichment of sgRNAs and genes was analyzed using MAGeCK (Version 0.5.9.2)^69^ by comparing read counts from cells after AMA selection with counts from matching unselected cell populations.

### Gene ontology (GO) and pathway enrichment analysis

GO terms in the screen were analyzed using DAVID (https://david.ncifcrf.gov/summary.jsp). KEGG pathways were analyzed using Webgestalt (http://www.webgestalt.org/). Enrichment network pathways were generated using the String (https://cn.string-db.org/) and Cytoscape (https://cytoscape.org/).

### Establishment of knockout cell lines

sgRNAs from the parent library were cloned into pLentiCRISPRv2 (# 52961, Addgene). The control sgRNA was used from the parent library (Supplementary Table 1). Lentiviruses were produced as described above and transduced HAP1 or HepG2 cells were selected with puromycin 24 h post-infection. After 7 days, puromycin was removed, and cells were allowed to recover for three additional days before analysis.

### RNA extraction and quantitative real-time PCR analysis

Total RNA was extracted using an RNA Quick Purification kit (#RN001, YIBIN). The cDNA was synthesized from 500 ng of total RNA by PrimeScript RT Master Mix (#RR037A, Takara) according to the manufacturer’s instructions. Quantitative PCR (qPCR) was performed with TB green (#RR820A, Takara) according to the manufacturer’s protocol with LightCycler96 (Roche).

### High-performance liquid chromatography (HPLC)

HPLC analysis was performed using SHIMADZU LC-20AT and a 250 mm  ×  4.6 mm liquid chromatography column (5  μm, Phenomenex). Ammonium acetate (50 mM, pH 5.5 acetic acid), acetonitrile, and methanol (80/10/10; v/v/v) were used as the mobile phase following a previous study^70^. An isocratic elution was performed at a flow rate of 1.0 mL/min, with 35 °C of column temperature. A total of 2 × 10^7^ cells treated with AMA for 8 h were collected and then broken by ultrasonication at 200 W (3 s work/ 3 s break) for 1 min on ice water. This mixture was centrifuged at 15000 g for 30 min. The supernatant was collected and filtered through a 0.22 μm filter (Merck Millipore). A total of 10 μL sample was injected into the HPLC system. For quantitative analyses, chromatograms were integrated at 303 nm. The peak area was analyzed by LabSolutions software (Version 1.26).

### Western blot analysis

Cells were harvested in a lysis buffer (#FD009, Fdbio) containing a protease inhibitor cocktail (Roche). Total cell lysates were centrifuged at 15000 g for 10 min at 4 °C to remove cell debris. The protein concentrations were determined using the BCA Protein Assay (#P0010, Beyotime). The protein supernatants were mixed with 5×loading buffer (FD006, Fdbio) and heated for 10 min at 100 °C. The proteins (20 μg) were electrophoresed on 10% SDS-polyacrylamide gels, transferred to PVDF membranes, and incubated with specific primary antibodies at 4 °C overnight. After washing, the membranes were incubated with horseradish peroxidase (HRP)-conjugated secondary antibodies for 1 h. Immunoblots were visualized by the ChemiDoc^TM^ imaging system (Bio-Rad, Version 2.4.0.03) using the enhanced chemiluminescent substrate (FD8000, Fdbio).

For protein detection, the following antibodies were used: mouse monoclonal antibody recognizing OATP1B3 (#66381-1-Ig, 1:5000, CloneNo.1D9A4), mouse monoclonal antibody recognizing GRP78(#66574-1-Ig, 1:5000, CloneNo.1D6F7), rabbit polyclonal antibody recognizing IRE1(#27528-1-AP, 1:1000), mouse monoclonal antibody recognizing GM130(#66662-1-Ig, 1:5000, CloneNo.2A4F11), rabbit polyclonal antibody recognizing ARF4(#11673-1-AP, 1:1000) were purchased from Proteintech. Rabbit polyclonal antibody recognizing NTCP (#ABP53103, 1:1000) was purchased from Abbkine. Mouse antibody recognizing β-tubulin (#FD0064, 1:5000) was purchased from Fdbio. Goat anti-mouse IgG(H + L) HRP (#BS12478, 1:5000) and goat anti-rabbit IgG(H + L) HRP (#BS13278, 1:5000) were purchased from Bioworld.

### In silico screen of FDA-approved drugs

The two libraries of FDA-approved compounds were downloaded from ZINC (1615 ligands) and Drugbank (1586 ligands) respectively. The chemical database was energy-minimized using MMFF94 force field (steepest descent) in the open-source OpenBabel software package (http://openbabel.org/) and saved in mol2 format. The compound library is then pre-processed and saved in pdbqt format using prepare_ligand4.py from AutoDock tools(https://ccsb.scripps.edu/mgltools/).

The crystal structure of STT3B was obtained from RCSB PDB (PDB ID: 6S7T)^71^. The STT3B was pre-processed by PyMol (http://pymol.org/2/) to remove water and ligands, by AutoDock tools to add polar hydrogens and Kollman charge. After these processes, STT3B was then saved in pdbqt format. There are two binding pockets in STT3B. The binding site grid box was visually defined by employing AutoDock tools’ Grid setting feature. The grid box of two binding pockets was defined by bound peptide and bound dolichylphosphate in 6S7T. For the STT3B pocket 1, the grid size dimensions were 23.25 × 22.5 × 24, with the (173.747, 153.363, 152.254) point set as the center coordinates; for the STT3B pocket 2, the grid size dimensions were 23.25 × 24.75 × 24.75, with the (159.012, 143.831, 166.532) point set as the center coordinates.

The docking procedure was performed with Smina (a fork of AutoDock Vina, https://vina.scripps.edu/)^72^. Molecular docking parameters are used as follows: exhaustiveness = 8, num_modes = 10, energy_range = 3, min_rmsd_filter = 1. A stepwise screening of FDA-approved drugs in the ZINC and Drugbank databases was performed. First, the compounds were docked with the two binding pockets of STT3B, respectively. Then the compounds were ranked by their minimized affinity. The top 100 minimized affinity ligands were obtained. Subsequently, the predicted IC_50_ value of the compounds was calculated by the neural-network-based-scoring function (NNScore2)^73^. In the end, the top 34 compounds were selected for in vitro validation. Due to the unavailability of some compounds, only 24 compounds were tested for cellular protection against AMA toxicity.

### Organoids culture

The mice liver organoids were generated according to previously described protocols with some modifications^74,75^. Briefly, the livers of CD-1 mice (20–30 g) were dissected into cubes of 1–2 mm^3^ and washed twice in PBS. The tissue fragments were incubated in digestion buffer (1 mg/mL Collagenase I, 0.1 mg/mL Hyaluronidase, 0.1 mg/mL DNase I) for 1.5 h at 37 °C. After digestion, the tissue suspension was filtered with a 40 μm cell strainer and then centrifuged and resuspended with PBS. Single-cell suspensions were initially seeded at high density and reseeded at a lower density after ∼1 week in complete organoid medium (DMEM/F12 with 5 μg/mL insulin, 250 ng/mL amphotericin B, 10 μg/mL gentamicin, 0.125 ng/mL EGF, 25 ng/mL hydrocortisone, and 10 μm Y-27632).

### Intracellular localization analysis

HAP1 and HepG2 cells were plated into the culture dish until ~50% confluence and incubated with ICG (#H20055881, Dandong Pharmaceutical Company) for 12 h. Cells were washed with PBS three times and ER was labeled with ER-tracker green according to the manufacturer’s protocol (#C1042S, Beyotime), and the nucleus was stained blue by 4′,6-diamidino-2-phenylindole (DAPI). Fluorescence images were obtained by confocal laser scanning microscopy.

### Luciferase reporter assay

The ER-LucT sequence and LucT sequence from a previous study^49^ were synthesized and cloned into pcDNA3.1(+) by Kidan Bio Co. Ltd. Plasmid transfections were performed with Lipo8000^TM^. After 48 h, ICG was added to transfected HAP1 and HepG2 cells. Luciferase activity assays were performed using the Firefly Luciferase Reporter Gene Assay Kit according to the manufacturer’s protocol (#RG005, Beyotime) with the Luminescence Quick Read (Promega).

### Mice

All mice were male CD-1 mice weighing 20–30 g (4–5 weeks old) and were purchased from the Laboratory Animal Center of Sun Yat-Sen University. Mice were maintained in a specific pathogen-free facility with controlled temperature (23 ± 2 °C), humidity (50–65%), and light/dark cycle of 12 h/12 h. Mice were free to access food and water.

Body weight, motor activity, dyspnea, and overall welfare of the animals were daily observed. Mice were euthanized according to the IACUC approved methods of anesthesia with 1% pentobarbital sodium followed by cervical dislocation.

### Short‑term mouse study (24 h)

Mice were randomly distributed into 4 groups (*n* = 6) and treated as follows: (i) control group (0.9% NaCl, i.p. at 0, 4, 8, and 12 h); (ii) ICG group (0.9% NaCl, i.p. at 0 h; 5 mg/kg ICG, i.v. at 4, 8 and 12 h); (ii) AMA group (0.33 mg/kg AMA i.p. at 0 h; 0.9% NaCl, i.v. at 4, 8, and 12 h); (iii) AMA + ICG group, (0.33 mg/kg AMA i.p. at 0 h; 5 mg/kg ICG i.p. at 4, 8, and 12 h).

### Long-term mouse study (30 days)

Mice were randomly divided into 4 groups (*n* = 6) and submitted to the same administration protocol as that of the short-term study. Body weight, motor activity, dyspnea, and overall welfare of the animals were daily observed for 30 days.

### In vivo fluorescence imaging

A total of 5 mg/kg ICG was intravenously injected and images were taken at 0, 10, 20, 40, 60, and 120 min after injection using an in vivo imaging system (PerkinElmer).

### Blood biomarkers

After 24 h of the AMA injection, all animals were anesthetized and euthanized. Blood was taken into EDTA-containing tubes and immediately centrifuged at 3000 g for 10 min (4 °C). The plasma supernatant was collected into tubes and stored at −80 °C until determination. AST, ALT, ALP, BUN, and Cre were measured by Guangdong Engineering & Technology Research Center for Disease-Model Animals, Sun Yat-sen University.

### Histological analysis of liver and kidney

After blood collection, the liver and kidneys were removed and weighed. Segments of the liver and kidney were placed in 4% paraformaldehyde for H&E staining by Servicebio technology. Inflammation and necrosis degree of slides were semi-quantified in a blind fashion according to the following criterion: Grade 0 = no change from normal; Grade 1 = very mild (The changes are just outside the normal range); Grade 2 = mild (Lesions can be observed, but they are not serious); Grade 3 = middle (The lesions are obvious and likely to be more severe) and Grade 4 = severe (the lesion has occupied the entire tissue and organ).

### Lectin staining

Liver sections from different groups of mice were cut, deparaffinized, and stained by diluted lectins for 30 min at room temperature. Fluorescein-SNA (binds sialic acid, FL-1301-2, 1:200) and Fluorescein-PHA-L (binds complex glycans, #FL-1111-2, 1:200) were purchased from Vector Laboratories. The slides were then stained with DAPI and washed twice in PBST (Phosphate Buffered Saline with Triton X-100) and imaged using fluorescence microscopy (Nikon).

### Statistics and reproducibility

No statistical method was used to predetermine the sample size. No data were excluded from the analyses. Data are presented as the mean ± standard deviation (S.D.). Statistical analyses were performed using GraphPad Prism 9 software (Version 9.0.0). *p* < 0.05 was considered statistically significant. *p < 0.05; **p < 0.01; ***p < 0.001; ****p < 0.0001; ns, not significant. Biological replicates and numbers of independent experiments were stated in the legends. All experiments presented as representative micrographs or gels were repeated at least 3 times with similar results.

### Reporting summary

Further information on research design is available in the Nature Portfolio Reporting Summary linked to this article.

### Supplementary information

Supplementary Information

Description of Additional Supplementary Files

Supplementary Dataset 1

Supplementary Dataset 2

AMA ChemDraw

Reporting Summary

### Source data

Source data

## Data Availability

DNA sequencing data generated in this study have been deposited in the Gene Expression Omnibus (GEO) database under accession code GSE226447 (https://www.ncbi.nlm.nih.gov/geo/query/acc.cgi?acc=GSE226447). ZINC (https://zinc20.docking.org/) and Drugbank (https://go.drugbank.com/) are publicly available datasets. The data supporting the findings of this study are available within the article and Supplementary Information file. Source data are provided in this paper. Source data are provided with this paper.
