# Supplementary material for: Identification of indocyanine green as a STT3B inhibitor against mushroom α-amanitin cytotoxicity
Source: Nat Commun. 2023 May 16;14:2241. doi: 10.1038/s41467-023-37714-3 (PMC10188588; doi:10.1038/s41467-023-37714-3)
Supplement: Supplementary file 1 — Supplementary Information [file 41467_2023_37714_MOESM1_ESM.pdf]

Supplementary Information

Identification of indocyanine green as a STT3B inhibitor against mushroom α-amanitin cytotoxicity

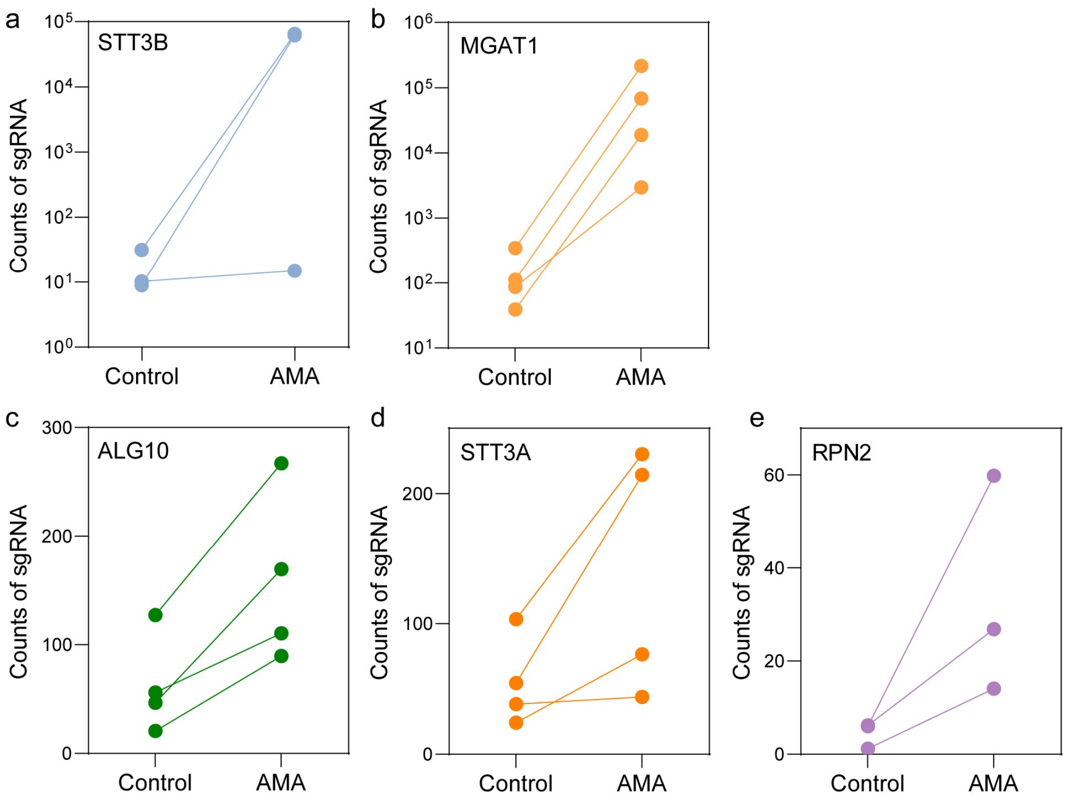

Supplementary Figure 1. **The gene hits from genome-wide CRISPR screen against AMA-induced cell death were enriched in the N-Glycan biosynthesis pathway.** The counts of sgRNA of five significant genes, *STT3B*(**a**), *MGAT1*(**b**), *ALG10*(**c**), *STT3A*(**d**), and *RPN2*(**e**), on the N-Glycan biosynthesis pathway. The sgRNAs targeting the five genes were consistently enriched in AMA-treated cells. Source data are provided as a Source Data file.

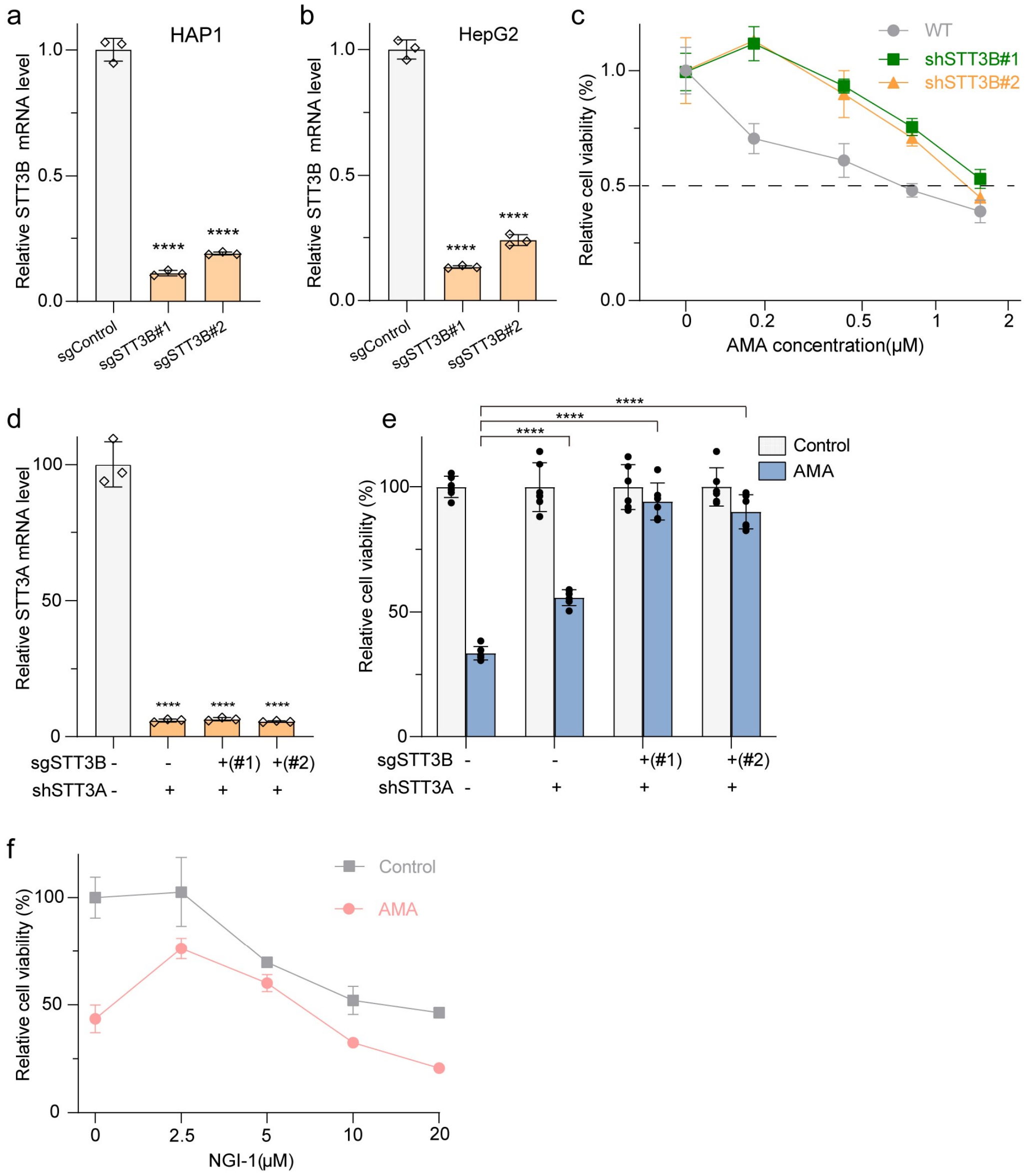

Supplementary Figure 2. **N-Glycan biosynthesis is essential for AMA-induced cell death. a-b** Relative *STT3B* mRNA level in *STT3B* knockout HAP1 (**a**) and HepG2 (**b**) cells (n = 3 biological replicates). ****p <0.0001. **c** WT and shSTT3B HepG2 cells were treated with vehicle or AMA for 72 h, and cell viability was determined by CCK8 assay (n = 3 biological replicates). **d-e** The combination of *STT3B* knockout and STT3A knockdown gave complete resistance to AMA in HepG2 cells. (**d**) The STT3A mRNA level of shSTT3A in sgSTT3B HepG2 cells (n = 3 biological replicates). ****p <0.0001. (**e**) Knockdown of *STT3A* with *STT3B* knockout confers complete resistance to AMA (5 μM) in HepG2 cell lines (n = 6 biological replicates). ****p <0.0001. **f** HAP1 cells were pre-treated with NGI for 12 h, and then treated with AMA (3 μM) for 48 h (n = 3

biological replicates). Data are presented as mean ± S.D. and are representative of three independent experiments. The statistics were assessed using one-way ANOVA followed by Dunnett's multiple comparisons test. Source data are provided as a Source Data file.

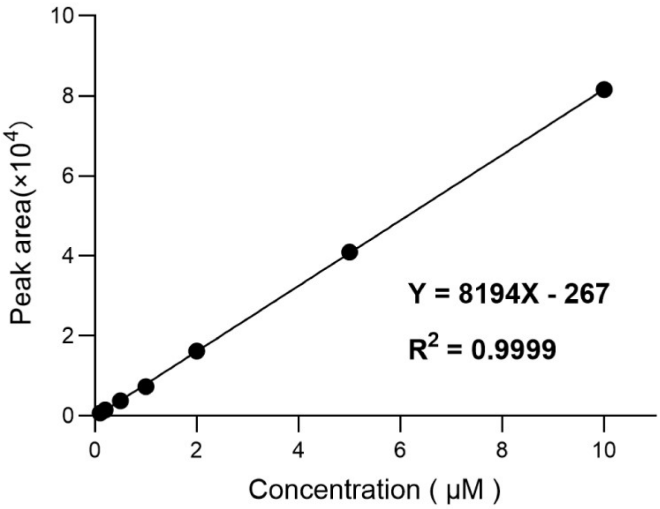

Supplementary Figure 3. The calibration curve of AMA concentration (0.1-10 µM) and peak area. The calibration curve showed a good linear relationship between AMA concentration (0.1-10 µM) and peak area, with R^2 = 0.9999. Source data are provided as a Source Data file.

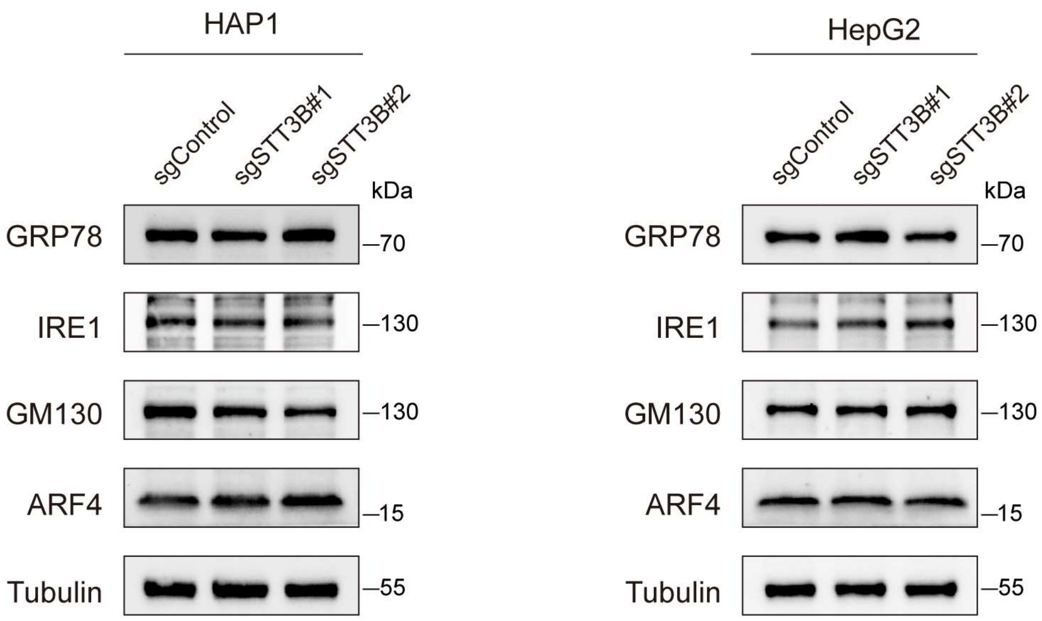

Supplementary Figure 4. **The knockout of *STT3B* did not cause a significant change in ER and Golgi stress markers**. Western blot analysis of stress response of ER (GRP78 and IRE1) and Golgi (GM130 and ARF4) in *STT3B* knockout cells. Source data are provided as a Source Data file.

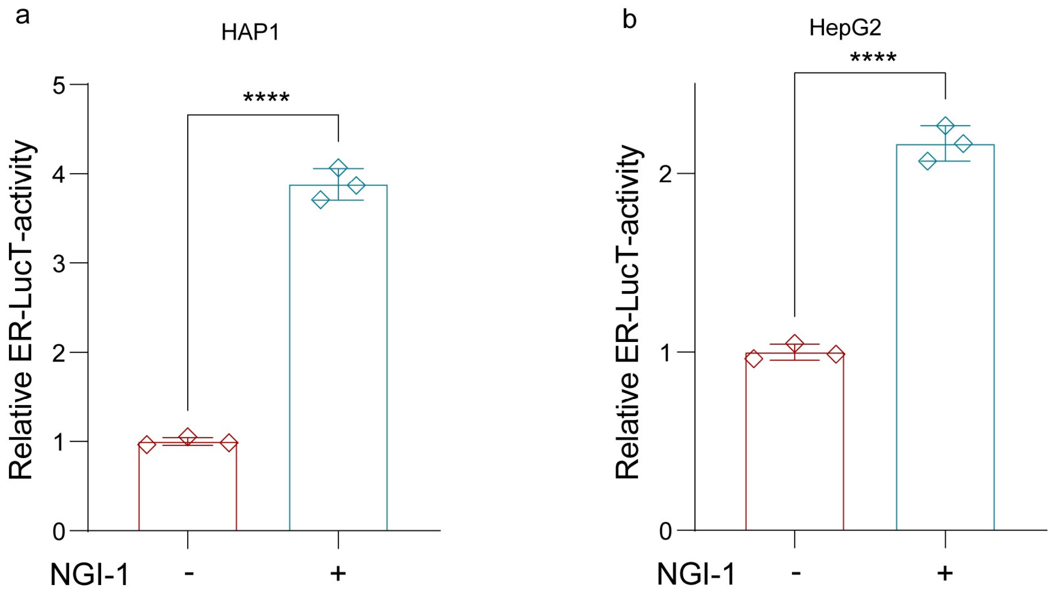

Supplementary Figure 5. The effect of NGI-1 on disrupting N-linked glycosylation. ERLucT transfected HAP1 (**a**) and HepG2 (**b**) cells were treated with NGI-1 at 10 µM and 20 µM, respectively, and luciferase activity was measured (n = 3 biological replicates). ****p <0.0001. The statistics were assessed using a two-tailed unpaired t test. Data are presented as mean ± S.D. and are representative of three independent experiments. Source data are provided as a Source Data file.

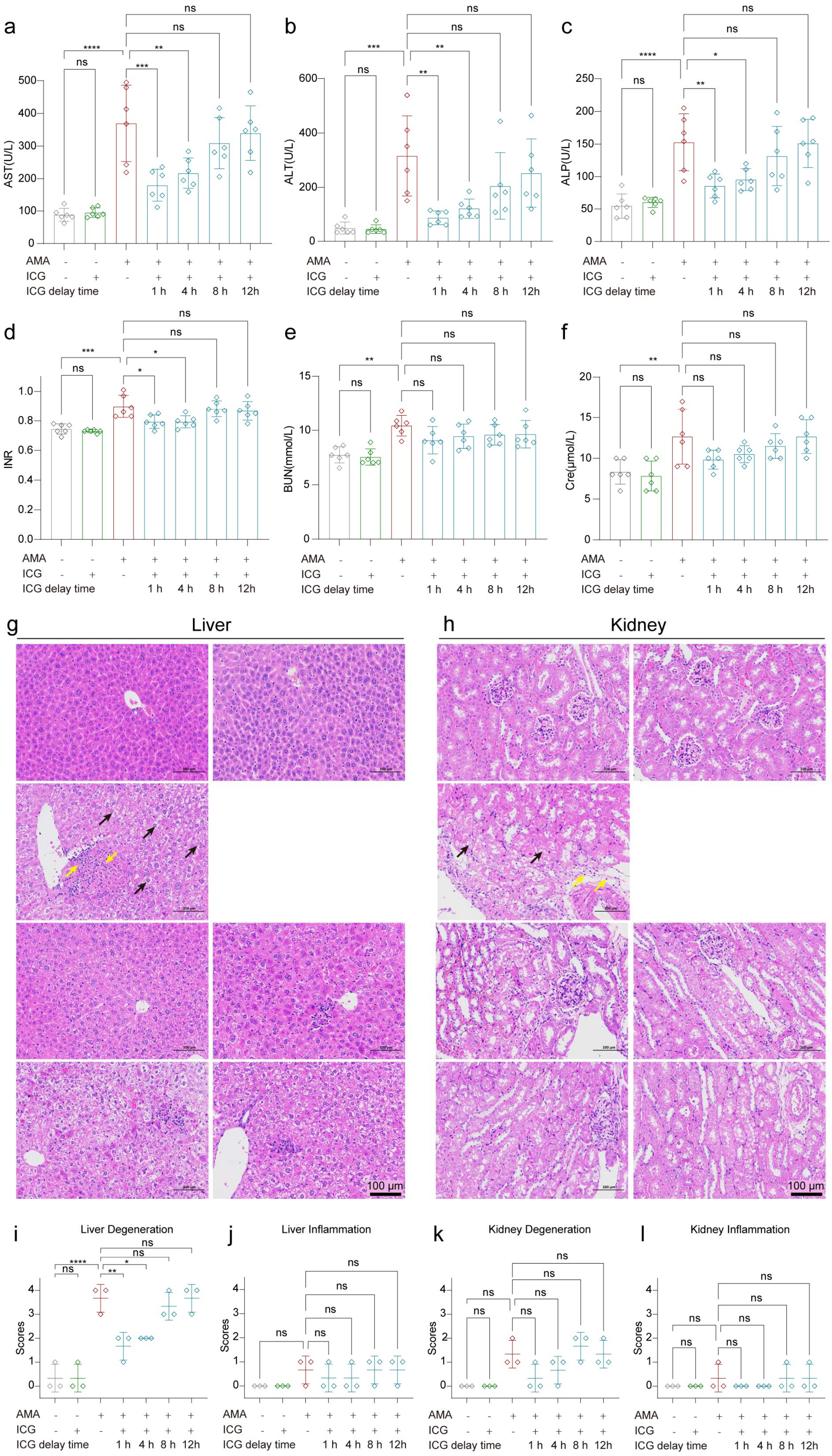

a
AST(U/L)
b
ALT(U/L)
c
ALP(U/L)
d
INR
e
BUN(mmol/L)
f
Cre(μmol/L)
AMA
ICG
ICG delay time
1 h
4 h
8 h
12h
ns
g
Liver
h
Kidney
100 μm
i
Liver Degeneration
j
Liver Inflammation
k
Kidney Degeneration
l
Kidney Inflammation
Scores

Supplementary Figure 6. ICG is an effective antidote for AMA toxicity in mice. a-f Plasma levels of AST, ALT, ALP, INR, BUN, Cre in mice with different treatments (n = 6 biological replicates). (**a**) $^{ns}p > 0.9999$, $^{****}p < 0.0001$, $^{***}p = 0.0005$, $^{**}p = 0.0067$, $^{ns}p = 0.7106$, $^{ns}p = 0.9865$; (**b**) $^{ns}p > 0.9999$, $^{***}p = 0.0002$, $^{**}p = 0.0015$, $^{**}p = 0.0095$, $^{ns}p = 0.3460$, $^{ns}p = 0.8754$; (**c**) $^{ns}p = 0.9999$, $^{****}p < 0.0001$, $^{**}p = 0.0081$, $^{*}p = 0.0341$, $^{ns}p = 0.8868$, $^{ns}p > 0.9999$; (**d**) $^{ns}p = 0.9984$, $^{***}p = 0.0001$, $^{*}p = 0.0149$, $^{*}p = 0.0149$, $^{ns}p = 0.9972$, $^{ns}p = 0.9428$; (**e**) $^{ns}p = 0.9999$, $^{**}p = 0.0011$, $^{ns}p = 0.2863$, $^{ns}p = 0.6525$, $^{ns}p = 0.7902$, $^{ns}p = 0.8318$; (**f**) $^{ns}p = 0.9993$, $^{**}p = 0.0072$, $^{ns}p = 0.1764$, $^{ns}p = 0.4676$, $^{ns}p = 0.9393$, $^{ns}p > 0.9999$. **g-h** H&E staining of liver and kidney in mice with different treatments. Cell degeneration (black arrow) and inflammatory cells (yellow arrow) were shown. Scale bars are 100 μm. **i-l** Pathological score of H&E staining of liver and kidney in different groups (n = 3 biological replicates). (**i**) $^{ns}p > 0.9999$, $^{****}p < 0.0001$, $^{**}p = 0.0060$, $^{*}p = 0.0241$, $^{ns}p = 0.9850$, $^{ns}p > 0.9999$; (**j**) $^{ns}p > 0.9999$, $^{ns}p = 0.6423$, $^{ns}p = 0.9764$, $^{ns}p = 0.9764$, $^{ns}p > 0.9999$, $^{ns}p > 0.9999$; (**k**) $^{ns}p > 0.9999$, $^{ns}p = 0.0564$, $^{ns}p = 0.2270$, $^{ns}p = 0.6423$, $^{ns}p = 0.9764$, $^{ns}p > 0.9999$; (**l**) $^{ns}p > 0.9999$, $^{ns}p = 0.9241$, $^{ns}p = 0.9241$, $^{ns}p = 0.9241$, $^{ns}p > 0.9999$, $^{ns}p > 0.9999$. All data were represented as mean ± S.D. The statistics were assessed using one-way ANOVA followed by Tukey's multiple comparisons test. Source data are provided as a Source Data file.

Supplementary Table 1. The various sequences used in this study.

Primer sequences for amplifying sgRNA library	
Sense	TTGTGGAAAGGACGAAACACCG
Antisense	CCAATTCCCACTCCTTTCAAGACCT
sequences used for gene validation	
sgSTT3B#1	CCAGGGTTGATGATAACCGC
sgSTT3B#2	AAGAAAGACACCCAAGTCGT
sgNT	GGATCTAGCTACCTCAAAAG
shSTT3B#1-F	CCGGGCACTTCAGTTCACATACTATCTCGAGATAGTATGTGAACTGAAGTGCTTTTTG
shSTT3B#1-R	AATTCAAAAAGCACTTCAGTTCACATACTATCTCGAGATAGTATGTGAACTGAAGTGC
shSTT3B#2-F	CCGGGCAGTATCTGAGAGACCGATTCTCGAGAATCGGTCTCTCAGATACTGCTTTTTG
shSTT3B#2-R	AATTCAAAAAGCAGTATCTGAGAGACCGATTCTCGAGAATCGGTCTCTCAGATACTGC
shSTT3A-F	CCGGCGTCATAATACTCCAGAGGATCTCGAGATCCTCTGGAGTATTATGACGTTTTTG
shSTT3A-R	AATTCAAAAACGTCATAATACTCCAGAGGATCTCGAGATCCTCTGGAGTATTATGACG
Real-time RT-PCR primer	
STT3B-F	AGTAGGTGGTACTGTTTACCCAG
STT3B-R	AAGTTGGTGCAAGGAACACAC
STT3A-F	GAAGCAACAGGATTCCACCTACC
STT3A-R	CAATGGACGGAGAAGAGTAGGC
GAPDH-F	GGAGCGAGATCCCTCCAAAAT
GAPDH-R	GGCTGTTGTCATACTTCTCATGG
