# Supplementary material for: Identification of indocyanine green as a STT3B inhibitor against mushroom α-amanitin cytotoxicity
Source: Nat Commun. 2023 May 16;14:2241. doi: 10.1038/s41467-023-37714-3 (PMC10188588; doi:10.1038/s41467-023-37714-3)
Supplement: Supplementary file 2 — Description of Additional Supplementary Files [file 41467_2023_37714_MOESM2_ESM.pdf]

Description of Additional Supplementary Files

File Name: Supplementary Data 1.
Description: The list of gene hits and scores from the genome-wide CRISPR screen against α-amanitin toxicity.

File Name: Supplementary Data 2.
Description: The results of molecular docking between STT3B and FDA-approved drug libraries.
