# Supplementary material for: Identification of indocyanine green as a STT3B inhibitor against mushroom α-amanitin cytotoxicity
Source: Nat Commun. 2023 May 16;14:2241. doi: 10.1038/s41467-023-37714-3 (PMC10188588; doi:10.1038/s41467-023-37714-3)
Supplement: Supplementary file 6 — Reporting Summary [file 41467_2023_37714_MOESM6_ESM.pdf]

nature portfolio

Corresponding author(s): Professor Qiaoping Wang

Last updated by author(s): Mar 2, 2023

Reporting Summary

Nature Portfolio wishes to improve the reproducibility of the work that we publish. This form provides structure for consistency and transparency in reporting. For further information on Nature Portfolio policies, see our Editorial Policies and the Editorial Policy Checklist.

Statistics

For all statistical analyses, confirm that the following items are present in the figure legend, table legend, main text, or Methods section.

n/a	Confirmed	
☐	☒	The exact sample size (*n*) for each experimental group/condition, given as a discrete number and unit of measurement
☐	☒	A statement on whether measurements were taken from distinct samples or whether the same sample was measured repeatedly
☐	☒	The statistical test(s) used AND whether they are one- or two-sided<br>*Only common tests should be described solely by name; describe more complex techniques in the Methods section.*
☒	☐	A description of all covariates tested
☐	☒	A description of any assumptions or corrections, such as tests of normality and adjustment for multiple comparisons
☐	☒	A full description of the statistical parameters including central tendency (e.g. means) or other basic estimates (e.g. regression coefficient) AND variation (e.g. standard deviation) or associated estimates of uncertainty (e.g. confidence intervals)
☐	☒	For null hypothesis testing, the test statistic (e.g. *F*, *t*, *r*) with confidence intervals, effect sizes, degrees of freedom and *P* value noted<br>*Give P values as exact values whenever suitable.*
☒	☐	For Bayesian analysis, information on the choice of priors and Markov chain Monte Carlo settings
☒	☐	For hierarchical and complex designs, identification of the appropriate level for tests and full reporting of outcomes
☒	☐	Estimates of effect sizes (e.g. Cohen's *d*, Pearson's *r*), indicating how they were calculated

Our web collection on statistics for biologists contains articles on many of the points above.

Software and code

Policy information about availability of computer code

Data collection	no software was used for data collection
Data analysis	MAGeCK (Version 0.5.9.2) was used to compare read counts.<br>GO terms were analyzed using DAVID (https://david.ncifcrf.gov/summary.jsp).<br>KEGG pathways were analyzed using Webgestalt (http://www.webgestalt.org/).<br>Enrichment network pathways were generated using the String (https://cn.string-db.org/) and Cytoscape (https://cytoscape.org/).<br>The peak area was analyzed by LabSolutions software (Version 1.26).<br>IImmunoblots were visualized by the ChemiDocTM imaging system (Bio-Rad, Version 2.4.0.03).<br>The chemical database was energy-minimized in open-source OpenBabel software package (http://openbabel.org/) .<br>The STT3B was pre-processed by PyMol (http://pymol.org/2/) .<br>The docking procedure was performed with Smina (a fork of AutoDock Vina, https://vina.scripps.edu/).<br>GraphPad Prism 9.0.0 and Image J 1.53 were used to analyze data.

For manuscripts utilizing custom algorithms or software that are central to the research but not yet described in published literature, software must be made available to editors and reviewers. We strongly encourage code deposition in a community repository (e.g. GitHub). See the Nature Portfolio guidelines for submitting code & software for further information.

Data

Policy information about availability of data

All manuscripts must include a data availability statement. This statement should provide the following information, where applicable:

- Accession codes, unique identifiers, or web links for publicly available datasets
- A description of any restrictions on data availability
- For clinical datasets or third party data, please ensure that the statement adheres to our policy

DNA sequencing data generated in this study have been deposited in the Gene Expression Omnibus (GEO) database under accession code GSE226447 (https://www.ncbi.nlm.nih.gov/geo/query/acc.cgi?acc=GSE226447). ZINC (https://zinc20.docking.org/) and Drugbank (https://go.drugbank.com/) are publicly available datasets. The data supporting the findings of this study are available within the article and Supplementary Information file. Source data are provided in this paper.

Human research participants

Policy information about studies involving human research participants and Sex and Gender in Research.

Reporting on sex and gender	NA
Population characteristics	NA
Recruitment	NA
Ethics oversight	NA

Note that full information on the approval of the study protocol must also be provided in the manuscript.

Field-specific reporting

Please select the one below that is the best fit for your research. If you are not sure, read the appropriate sections before making your selection.

☒ Life sciences ☐ Behavioural & social sciences ☐ Ecological, evolutionary & environmental sciences

For a reference copy of the document with all sections, see nature.com/documents/nr-reporting-summary-flat.pdf

Life sciences study design

All studies must disclose on these points even when the disclosure is negative.

Sample size	No statistical method was used to predetermine the sample size. Biological replicates were stated in the legends.
Data exclusions	No data were excluded from the analyses.
Replication	All experiments were reproduced and independent numbers of experiments were stated in the legends.
Randomization	Cells plated in different well were randomly allocated into control and treatment groups. Quantifications from randomly selected imaging fields per experimental condition replicate. Animals are randomly assigned for each treatment groups.
Blinding	The investigator and personnel were not blinded during this study all experiments were performed based on standardized protocols and readouts and are not influenced by the investigator.

Reporting for specific materials, systems and methods

We require information from authors about some types of materials, experimental systems and methods used in many studies. Here, indicate whether each material, system or method listed is relevant to your study. If you are not sure if a list item applies to your research, read the appropriate section before selecting a response.

Materials & experimental systems

n/a	Involved in the study
☐	☒ Antibodies
☐	☒ Eukaryotic cell lines
☒	☐ Palaeontology and archaeology
☐	☒ Animals and other organisms
☒	☐ Clinical data
☒	☐ Dual use research of concern

Methods

n/a	Involved in the study
☒	☐ ChIP-seq
☒	☐ Flow cytometry
☒	☐ MRI-based neuroimaging

Antibodies

Antibodies used

For protein detection, the following antibodies were used: mouse monoclonal antibody recognizing OATP1B3 (#66381-1-Ig, 1:5000, CloneNo.1D9A4), mouse monoclonal antibody recognizing GRP78(#66574-1-Ig, 1:5000, CloneNo.1D6F7), rabbit polyclonal antibody recognizing IRE1(#27528-1-AP, 1:1000), mouse monoclonal antibody recognizing GM130(#66662-1-Ig, 1:5000, CloneNo.2A4F11), rabbit polyclonal antibody recognizing ARF4(#11673-1-AP, 1:1000) were purchased from Proteintech. Rabbit polyclonal antibody recognizing NTCP (#ABP53103, 1:1000) was purchased from Abbkine. Mouse antibody recognizing β-tubulin (#FD0064, 1:5000) was purchased from Fdbio. Goat anti-mouse IgG(H+L) HRP (#BS12478, 1:5000) and goat anti-rabbit IgG(H+L) HRP (#BS13278, 1:5000) were purchased from Bioworld.

Validation

Commercial available Western blot antibodies were selected based on their suggested application and previous validation as described on the manufacturer's website and data sheets.
OATP1B3: https://www.ptgcn.com/Products/SLCO1B3-Antibody-66381-1-Ig.htm
NTCP: https://www.abbkine.cn/product/abp53103/
GRP78: https://www.ptgcn.com/products/GRP78,BIP-Antibody-66574-1-Ig.htm
IRE1: https://www.ptgcn.com/products/IRE1--ERN1-Antibody-27528-1-AP.htm
GM130: https://www.ptgcn.com/products/GM130-GOLGA2-Antibody-66662-1-Ig.htm
ARF4: https://www.ptgcn.com/products/ARF4-Antibody-11673-1-AP.htm
β-tubulin: http://www.fdbio.net/productinfo.php?id=235

Eukaryotic cell lines

Policy information about cell lines and Sex and Gender in Research

Cell line source(s)

HAP1 cells were obtained from Horizon Discovery. HEK293T and HepG2 cell lines were obtained from the American Type Culture Collection (ATCC).

Authentication

All cell lines were authenticated by Short Tandem Repeat (STR) profiling.

Mycoplasma contamination

All cell lines were routinely tested with mycoplasma free.

Commonly misidentified lines
(See ICLAC register)

None

Animals and other research organisms

Policy information about studies involving animals; ARRIVE guidelines recommended for reporting animal research, and Sex and Gender in Research

Laboratory animals

All mice were male CD-1 mice weighing 20-30 g (4-5 weeks old) and were purchased from the Laboratory Animal Center of Sun Yat-Sen University.

Wild animals

No wild animals were used in this study.

Reporting on sex

Only male mice were used in this study.

Field-collected samples

No Field-collected samples were used in this study.

Ethics oversight

All animal experiments were approved by the Institutional Animal Care and Use Committee (IACUS) of Sun Yat-Sen University (Approval number: SYSU-IACUC-2022-000469).

Note that full information on the approval of the study protocol must also be provided in the manuscript.
